# Extraction of volatile organic compounds from leaves of *Ambrosia artemisiifolia* L. and *Artemisia annua* L. by headspace-solid phase micro extraction and simultaneous distillation extraction and analysis by gas chromatography/mass spectrometry

**DOI:** 10.1007/s10068-020-00875-8

**Published:** 2021-02-19

**Authors:** Ji Hyeon Son, Md Atikul Islam, Joon Ho Hong, Ji Young Jeong, Ok Yeon Song, Hui Eun Kim, Naeem Khan, Nargis Jamila, Kyong Su Kim

**Affiliations:** 1 Department of Food and Nutrition, Chosun University, Gwangju, 61452 Republic of Korea; 2 Department of Chemistry, Hajee Mohammad Danesh Science and Technology University, Dinajpur, 5200 Bangladesh; 3 Nanobio Research Center, Jeonnam Bioindustry Foundation (JBF), Jeollanam-do, 57248 Jangsung-gun, South Korea; 4 Department of Chemistry, Kohat University of Science and Technology, Kohat, 26000 Khyber Pakhtunkhwa Pakistan; 5 Department of Chemistry, Shaheed Benazir Bhutto Women University, Peshawar, 25000 Khyber Pakhtunkhwa Pakistan

**Keywords:** *Ambrosia artemisiifolia* L. leaves, *Artemisia annua* L. leaves, Volatile organic compound, Headspace-solid phase micro extraction (HS-SPME), Simultaneous distillation extraction (SDE), Gas chromatography/mass spectrometry (GC/MS)

## Abstract

This study was designed to analyze the volatile organic compounds in the leaves of *Ambrosia artemisiifolia* L. and *Artemisia annua* L. from Korea. For extraction of volatile compounds, headspace-solid phase micro extraction (HS-SPME) and simultaneous distillation extraction (SDE) were applied and analyzed by gas chromatography/mass spectrometry (GC/MS). From the results, SDE extraction was found to give the highest concentration of volatile compounds with an average concentration of 1,237.79 mg/kg for *A. annua* L. leaves compared to 1,122.73 mg/kg by HS-SPME technique. A total of 116 volatile organic compounds were identified, including 76 similar volatile organic compounds detected by both the methods of extraction in leaves of subject species at varying concentrations. Among these 33 volatile organic compounds were reported for the first time from the subject plant species. Thus the present research findings extend the characterization of volatile organic compounds from leaves of *A. annua* L. and *A. artemisiifolia* L. species and reported some distinguishing compounds which may be used for their discrimination.

## Introduction

*Ambrosia artemisiifolia* L. (common ragweed) leaves are highly allergenic pollens, causing great agricultural losses (Molinaro et al., 2016). These have negative impact on the diversity of plant species, richness and the composition of their vegetation is a topic of on-going discussion (Sölter et al., 2012). This species is indigenous to North America (Basset and Crompton, 1975) but also found abroad in South Asia, such as South Korea (Kil et al., 2004) and China (Sang et al., 2011; Xie et al., 2010). The allopathic action of *A. artemisiifola* L. leaves has already been reported in literature, while knowledge of their volatile organic compounds was still lacking.

In comparison, *Artemisia annua* L. (sweet wormwood) is a medicinal herb; native to South East Asia, including South Korea, China, and India (Wu et al., 2017). This plant has been used for many centuries in the treatment of malaria, fever, flavouring of sprit, perfumes, folk medicine and industrial purposes (Bilia et al., 2008; Ma et al., 2007; Nekoei et al., 2012). Recently, *A. annua* L. leaves were found to be effective against human leukemia, tumour, small-cell lung carcinomas, and breast cancer carcinomas (Efferth and Willmar 2007). Due to the similar appearance, consumers and manufacturers are confused to separate *A. annua* L. leaves in the market from *A. artemisiifolia* L. leaves.

Headspace-solid phase micro extraction (HS-SPME) is a quick, solvent less, and simple method for analysis of volatile organic compounds (Holt, 2001; Kataoka et al., 2000; Zhang et al., 1994). This technique is a non-invasive and non-destructive that avoids solvent impurity contamination (Heath and Reineccius, 1986). It combines sampling, extraction, and concentration into a simple continuous process (Adam et al., 2005). In contrary, Likens and Nickerson developed simultaneous distillation–extraction (SDE) is a traditional and widely used method for extraction of volatile organic compounds (Islam et al., 2020). Gu et al. (2009) indicated that SDE has outstanding high performance and reproducibility compared to other conventional methods such as hydro distillation and steam distillation.

Some wild toxic plants are morphologically very similar to important medicinal plants. When such wild toxic plants are accidentally taken, instead of medicinal plants for different purposes, they cause many problems. According to Kim and Jang (2019), in South Korea, 42 patients faced abdominal pain after intake of *A. artemisiifolia* L. leaves instead of *A. annua* L. leaves. So, it was very important to understand the volatile organic compounds characteristics of leaves of both the subject species selected for current study and to find out the distinguishing marker compounds. From literature search it was come to know that HS-SPME and SDE techniques were not used to isolate volatile organic compounds from *A. artemisiifolia* L. leaves and also there was no comparative study found on the volatile organic compounds of *A. artemisiifolia* L. leaves and *A. annua* L. leaves. In the current analysis, therefore both HS-SPME and SDE techniques coupled to GC/MS analysis were applied to establish the comparative results of volatile organic compounds profile of *A. artemisiifolia* L. leaves and *A. annua* L. leaves. It was also attempted to report distinguishing marker volatile organic compounds which may be used for their identification.

## Materials and methods

### Sample collection

**Fig. 1:**
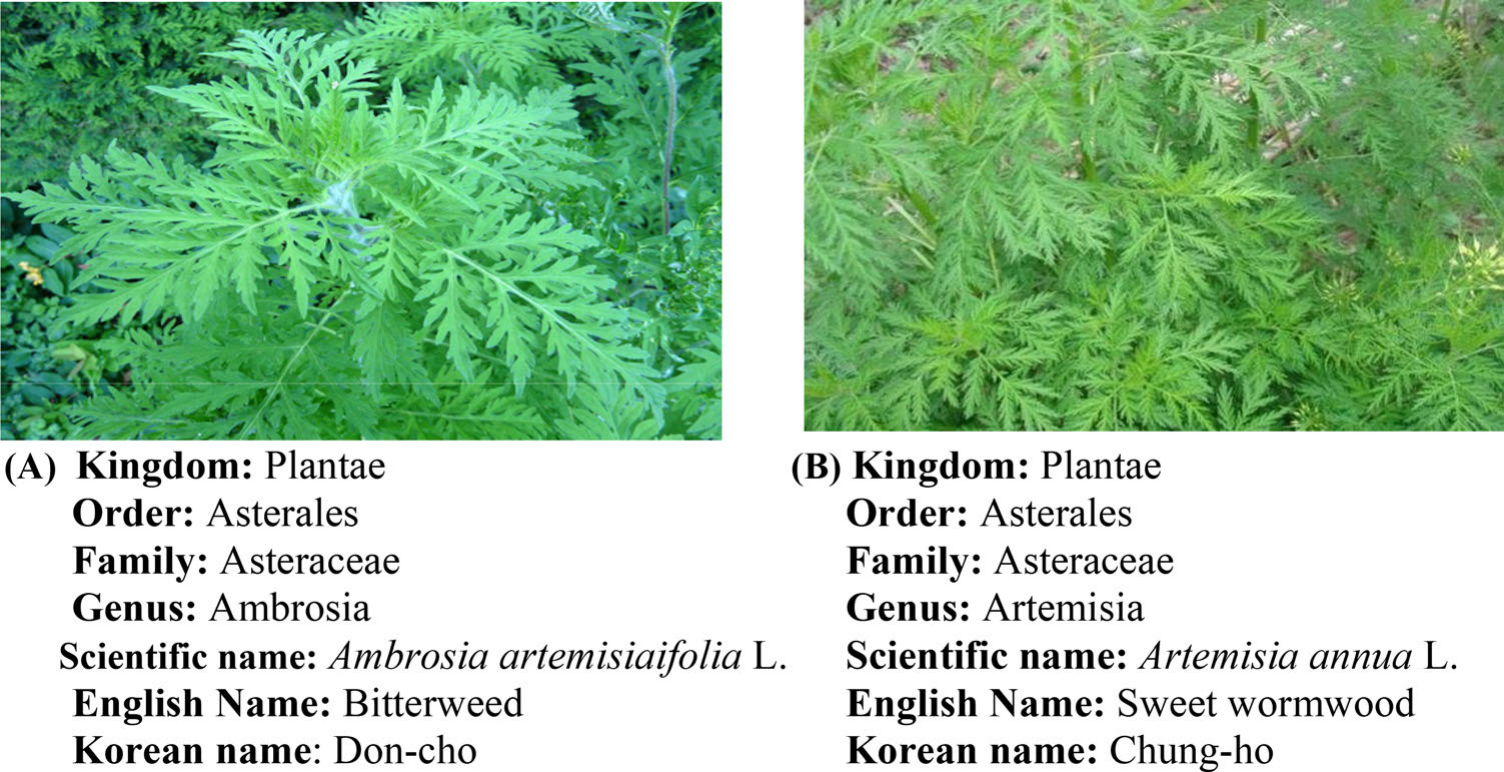
Picture and taxonomy of **(A)** *Ambrosia artemisiaifolia* L. leaves and **(B)** *Artemisia annua* L. leaves

Figures and taxonomy of the sampled plants are shown in Fig. 1. In February 2018, the authentic *A. artemisiifolia* L. leaves and *A. annua* L. leaves were collected in triplicate from Korea native plants growers farm association corporation, Seongnam-si, Republic of Korea These two leave varieties were thoroughly washed with clean water, followed by rinsing with distilled water. Then, using a blender (MR 350CA, Braun, Spain) the leaves were crushed, transferred to polythene bags, properly labelled and used for volatile compounds extraction and GCMS analysis.

#### Chemicals and reagents

All chemicals and reagents used in this study were purchased from Sigma-Aldrich Co. (St. Louis, MO, USA) and Thermo Fisher Scientific (Waltham, MA, USA). The water purification system (Millepore Corporation, Beds., USA) was used to prepare ultra-pure water. For the re-distillation of the organic solvents (*n*-pentane and diethyl ether), a wire spiral packed double distilling apparatus (Normschliff Geratebau, Wertheim, Germany) was used. Anhydrous sodium sulphate was burned overnight at 650 °C in furnace (F 6000, Barnsted Thermolyne Co., IA., USA) and then used for dehydration of organic solvent.

#### Extraction of volatile compounds using HS-SPME technique

Around 2.0 g of each homogenized sample of *A. artemisiifolia* L. leaves and *A. annua* L. leaves were taken into 15.0 mL headspace clear glass vials (Supelco, PA., USA) and then injected 50.0 µL (100 ppm in *n*-pentane) n-butyl benzene as an internal standard. After that, the headspace glass vial was hold on for 30 min in equilibrium temperature at 70 °C and then SPME fibre (50/30 µm DVB/CAR/PDMS, Supelco) was exposed for 5 min to absorb volatile organic compounds. Finally, the temperature of the injector was maintained at 250 °C, and the fibre was kept in GC/MS objector for 5 min to analyse the volatile organic compounds.

#### Extraction of volatile compounds using SDE technique

Every 30.0 g homogenized sample was mixed with 1,000 mL of distilled water and the pH was adjusted at 7.0 by dilute NaOH/HCl solution. For the quantitative analysis of volatile organic compounds, 100 ppm, 10 mL n-butyl benzene was added as an internal standard. Volatile organic compounds were extracted from both leaves by modified simultaneous distillation extraction (SDE, Likens & Nickerson types) apparatus with 100 mL redistilled n-pentane:diethyl ether (1:1, v/v) mixture. The experiment was maintained under normal atmospheric pressure for 3 h (Schultz et al., 1977). The SDE extract was dehydrated overnight by adding 10 g of anhydrous Na_2_SO_4_. Finally, the vigreux column was used to concentrate the extract volume to 1.5 mL. After that, it was again concentrated to 0.5 mL under N_2_ gas mild flush. Finally, the concentrated extract was injected into the GC/MS system for determination of volatile organic compounds (Khan et al., 2015).

#### Analysis of volatile organic compounds by GC/MS

The quantitative analysis of volatile organic compounds was carried out by Shimadzu GC/MS, QP-2010 (Shimadzu, Japan) with the EI (electron impact) mode. The ionization voltage was 70 eV, and temperatures of the injector and of ion source were maintained at 250 °C and 220 °C, respectively. The volatile organic compounds were isolated by GC column DB-5 (60 m × 0.25 mm i.d., film thickness 0.25 µm, Phenomenex, USA) and the mass spectra scanned were from 41 to 500 m/z. The GC/MS oven temperature was set as follows; 40 °C (5 min isothermal) raised to 220 °C at 2 °C/min and then to 280 °C at 10 °C/min (10 min isothermal). Helium gas at flow rate of 1.0 mL/min was used as the carrier gas. The sample injector volume was 1.0 µL and the split ratio was 1:20 (Islam et al., 2020).

#### Identification and quantification of volatile organic compounds

The *A. artemisiifolia* L. leaves and *A. annua* L. leaves volatile organic compounds were identified by the spectral databases, including NIST 12, FFNSC 2012, and WILEY 7. In addition, our own mass spectral database, and mass spectral data books were applied (Davies, 1990). Moreover, evaluations of retention indices to reference data were studied (Adams, 2007). The retention time of solutes of standard n-alkanes (C_8_–C_20_) mixture was used as an external reference (Jeon et al., 2017). The quantitative assessment of volatile organic compounds was carried out with the help of peak area percentage of the internal standard by using the formula:

$${\text{Volatile}} organic compounds amount \left( {mg/kg} \right) \, = \frac{{{\text{Pc}} \times 1000}}{{{\text{Pi}} \times {\text{As}} }}$$

where, Pi = Peak area (internal standard).

As = Amount of sample (g).

Pc = Peak area (component in sample).

## Statistical Analysis

The volatile organic compounds were calculated in triplicate, and the data was evaluated using the Software Version 20 (IBM, New York, USA) of Statistical Package for Social Sciences (SPSS). The final results were reported as mean ± standard deviation (mg/kg).

## Results and discussions

**Fig. 2:**
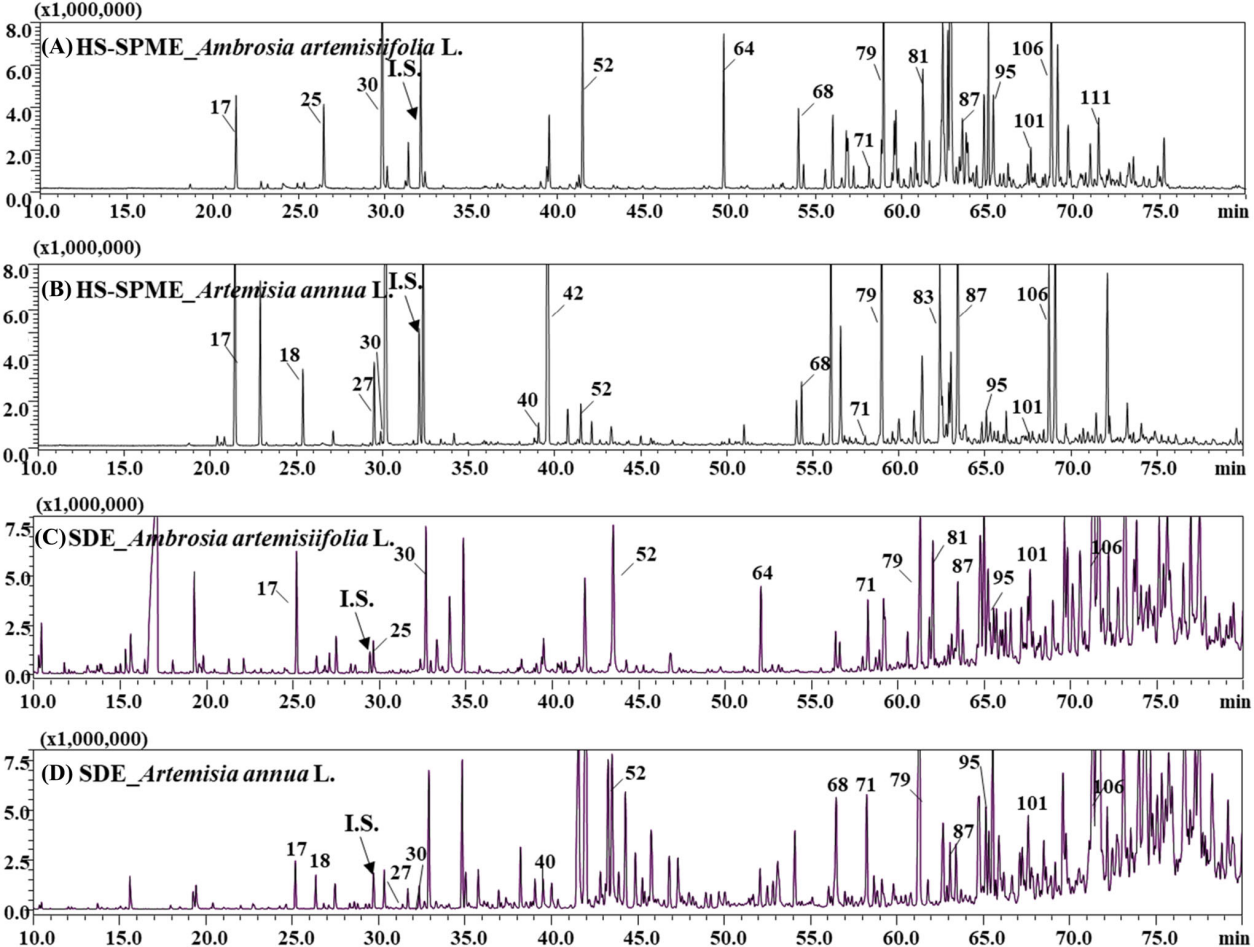
**(A)-(D)** Comparison of HS-SPME and SDE technique with GC/MS chromatograms of volatile compounds between *Ambrosia artemisiifolia* L. leaves and *Artemisia annua* L. leaves from South Korea. Peak information: (I.S) internal standard; (17) α-pinene; (18) camphene; (25) *β*-myrcene; (27) (*E,E*)-2,4-heptadienal; (30) limonene; (40) artemisa alcohol; (42) (*E*)-sabinene hydrate; (52) borneol; (64) bornyl acetate; (68) *α*-cubebene; (71) *α*-copaene; (79) *β*-caryophyllene; (81) (*Z*)- *α*-bergamotene; (83) coumarin; (87) *α*-caryophyllene; (95) *β*-selinene; (101) *δ*-cadinene; (106) caryophyllene oxide; (111) spatulenol

The GC/MS chromatograms of volatile compounds of various extracts from *A. artemisiifolia* L. leaves and *A. annua* L. leaves via HS-SPME and SDE techniques were as displayed in Fig. 2a–d. The identified volatile organic compounds detail such as retention index (RI), compound names, molecular formulae (MF), molecular weights (MW), peak areas %, and their concentrations were as shown in Table 1. From the results it was found that both the leaves contain a wide range of volatile organic compounds. In published literature, it has been reported that both the leaves contain around 30–35 compounds (Nekoei et al., 2012; Wang et al., 2006).

**Table 1:** Volatile compounds identified in *Ambrosia artemisiifolia* L. and *Artemisia annua* L. leaves samples

No	R.I^*^	Compound name	M.F^**^	M.W^***^	HS-SPME				SDE			
					*Ambrosia artemisiifolia* L		*Artemisia annua* L		*Ambrosia artemisiifolia* L		*Artemisia annua* L	
					Area (%)	(mg/kg)	Area (%)	(mg/kg)	Area (%)	(mg/kg)	Area (%)	(mg/kg)
1	706	**3-Methylbutanal**^**b**^	C_5_H_10_O	87	–	–	–	–	–	–	0.05 ± 0.00	0.68 ± 0.01
2	719	1-Pentene-3-ol^a^	C_5_H_10_O	86	–	–	–	–	–	–	0.05 ± 0.00	0.59 ± 0.00
3	727	Pentanal^b^	C_5_H_10_O	86	–	–	–	–	0.11 ± 0.00	1.25 ± 0.01	0.05 ± 0.01	0.62 ± 0.01
4	729	2-Ethylfuran^f^	C_6_H_8_O	96	–	–	–	–	–	–	0.09 ± 0.00	1.07 ± 0.02
5	754	2-Methyl-1-butanol^a^	C_5_H_12_O	88	–	–	–	–	–	–	0.03 ± 0.00	0.40 ± 0.01
6	767	(*E*)-2-Pentenal^b^	C_5_H_8_O	84	–	–	–	–	–	–	0.02 ± 0.00	0.25 ± 0.01
7	775	1-Pentanol^a^	C_5_H_12_O	88	–	–	–	–	0.16 ± 0.00	1.74 ± 0.01	0.08 ± 0.01	1.00 ± 0.02
8	794	2-Hexanone^e^	C_6_H_12_O	100	–	–	–	–	–	–	0.06 ± 0.00	0.71 ± 0.02
9	802	Hexanal^b^	C_6_H_12_O	100	0.15 ± 0.00	0.95 ± 0.04	0.24 ± 0.01	1.35 ± 0.01	0.14 ± 0.00	1.53 ± 0.01	0.52 ± 0.00	6.52 ± 0.10
10	836	Furfural^b^	C_5_H_4_O_2_	96	–	–	–	–	–	–	0.06 ± 0.00	0.74 ± 0.02
11	855	(*E*)-2-Hexenal^b^	C_6_H_10_O	98	–	–	–	–	0.17 ± 0.00	1.84 ± 0.00	0.29 ± 0.01	3.64 ± 0.04
12	857	(*Z*)-3-Hexen-1-ol^a^	C_6_H_12_O	100	–	–	–	–	0.15 ± 0.01	1.68 ± 0.01	0.45 ± 0.01	5.57 ± 0.06
13	871	**1-Hexanol**^**a**^	C_6_H_14_O	102	–	–	–	–	0.14 ± 0.00	1.57 ± 0.00	0.10 ± 0.01	1.23 ± 0.11
14	880	**Heptanal**^**b**^	C_7_H_14_O	114	–	–	–	–	–	–	0.04 ± 0.00	0.46 ± 0.04
15	894	**2-Heptanone**^**e**^	C_7_H_14_O	114	0.10 ± 0.00	0.65 ± 0.00	–	–	0.12 ± 0.01	1.35 ± 0.01	0.06 ± 0.01	0.74 ± 0.02
16	928	*α-*Thujene^e^	C_10_H_16_	136	–	–	–	–	–	–	0.09 ± 0.00	1.17 ± 0.12
17	935	*α-*Pinene^d^	C_10_H_16_	136	1.87 ± 0.00	12.19 ± 0.02	0.53 ± 0.02	2.91 ± 0.04	2.47 ± 0.02	27.52 ± 0.06	0.76 ± 0.02	9.44 ± 0.42
18	950	Camphene^d^	C_10_H_16_	136	0.15 ± 0.00	1.00 ± 0.00	0.49 ± 0.04	2.70 ± 0.08	–	–	0.53 ± 0.02	6.55 ± 0.32
19	956	2,4-Thujadiene^d^	C_10_H_14_	134	–	–	–	–	0.27 ± 0.00	2.96 ± 0.01	0.09 ± 0.01	1.16 ± 0.14
20	960	(*E*)-2-heptenal^b^	C_7_H_12_O	112	–	–	–	–	–	–	0.08 ± 0.02	1.01 ± 0.13
21	965	Benzaldehyde^b^	C_7_H_6_O	106	0.29 ± 0.02	1.88 ± 0.02	0.30 ± 0.01	1.65 ± 0.12	0.82 ± 0.02	9.17 ± 0.04	0.47 ± 0.02	5.79 ± 0.29
22	975	Sabinene^d^	C_10_H_16_	136	0.10 ± 0.00	0.67 ± 0.00	–	–	–	–	0.09 ± 0.00	1.12 ± 0.01
23	979	*β-*Pinene^d^	C_10_H_16_	136	0.12 ± 0.00	0.79 ± 0.00	–	–	–	–	0.11 ± 0.13	1.41 ± 0.04
24	990	**Sulcatone**^**e**^	C_8_H_14_O	126	–	–	–	–	0.40 ± 0.00	4.43 ± 0.03	–	–
25	992	*β-*Myrcene^d^	C_10_H_16_	136	1.80 ± 0.02	11.73 ± 0.08	–	–	0.68 ± 0.00	7.58 ± 0.02	–	–
26	1001	Yomogi alcohol^a^	C_10_H_18_O	154	–	–	0.52 ± 0.02	2.94 ± 0.04	–	–	0.65 ± 0.02	8.04 ± 0.51
27	1015	(*E,E*)-2,4-Heptadienal^b^	C_7_H_10_O	110	–	–	0.16 ± 0.00	0.90 ± 0.02	–	–	0.08 ± 0.01	1.05 ± 0.02
28	1019	*α-*Terpinene^d^	C_10_H_16_	136	–	–	–	–	–	–	0.32 ± 0.02	3.92 ± 0.14
29	1027	*p-*Cymene^d^	C_10_H_14_	134	–	–	0.27 ± 0.00	1.47 ± 0.01	–	–	0.37 ± 0.01	4.66 ± 0.21
30	1031	Limonene^d^	C_10_H_16_	136	3.89 ± 0.02	25.40 ± 0.04	0.13 ± 0.00	0.72 ± 0.02	3.33 ± 0.03	37.20 ± 0.11	0.18 ± 0.01	2.22 ± 0.24
31	1035	1,8-Cineole^f^	C_10_H_18_O	154	0.47 ± 0.00	3.04 ± 0.02	2.46 ± 0.02	13.56 ± 0.08	–	–	2.57 ± 0.01	31.91 ± 0.11
32	1040	Benzyl alcohol^a^	C_7_H_8_O	108	–	–	–	–	0.95 ± 0.02	10.60 ± 0.02	0.17 ± 0.02	2.12 ± 0.09
33	1049	**Benzene acetaldehyde**^**b**^	C_8_H_8_O	120	–	–	–	–	2.18 ± 0.02	24.37 ± 0.12	–	–
34	1050	**Phenyl acetaldehyde**^**b**^	C_8_H_8_O	120	0.17 ± 0.00	1.14 ± 0.00	–	–	–	–	–	–
35	1052	(*E*)-*β*-Ocimene^d^	C_10_H_16_	136	0.98 ± 0.02	6.42 ± 0.04	–	–	–	–	–	–
I.S	1059	*n*-Butylbenzene	C_10_H_14_	134	–	–	–	–	–	–	–	–
36	1062	*γ-*Terpinene^d^	C_10_H_16_	136	–	–	–	–	–	–	0.65 ± 0.02	8.08 ± 0.21
37	1063	Artemisia ketone^e^	C_10_H_16_O	152	0.39 ± 0.00	2.54 ± 0.02	–	–	–	–	–	–
38	1071	(*Z*)-Sabinene hydrate^a^	C_10_H_18_O	154	0.08 ± 0.00	0.55 ± 0.00	–	–	–	–	0.74 ± 0.01	9.23 ± 0.18
39	1074	1-Octanol^a^	C_8_H_18_O	130	–	–	–	–	–	–	0.07 ± 0.01	0.83 ± 0.02
40	1087	Artemisia alcohol^a^	C_10_H_18_O	154	–	–	1.75 ± 0.00	0.84 ± 0.02	–	–	0.30 ± 0.03	3.73 ± 0.13
41	1092	*α*-Terpinolene^d^	C_10_H_16_	136	–	–	–	–	–	–	0.36 ± 0.02	4.50 ± 0.11
42	1103	(*E*)-Sabinene hydrate^a^	C_10_H_18_O	154	–	–	0.13 ± 0.00	0.72 ± 0.04	–	–	1.13 ± 0.01	14.07 ± 0.30
43	1104	Linalool^a^	C_10_H_18_O	154	–	–	–	–	0.49 ± 0.00	5.51 ± 0.01	–	–
44	1120	Phenethyl alcohol^a^	C_8_H_10_O	122	–	–	–	–	–	–	0.74 ± 0.01	9.25 ± 0.20
45	1132	*α-*Campholenal^b^	C_10_H_16_O	152	–	–	–	–	–	–	0.21 ± 0.02	2.65 ± 0.04
46	1147	(*E*)-Pinocarveol^a^	C_10_H_16_O	152	–	–	1.31 ± 0.01	7.17 ± 0.01	–	–	5.27 ± 0.02	65.55 ± 6.41
47	1150	**(*E*)-Verbenol**^**a**^	C_10_H_16_O	152	0.40 ± 0.00	2.63 ± 0.02	–	–	2.62 ± 0.02	29.28 ± 0.08	–	–
48	1153	Camphor^e^	C_10_H_16_O	152	1.62 ± 0.02	10.56 ± 0 .04	11.8 ± 0.11	65.76 ± 0.12	–	–	10.66 ± 0.08	132.61 ± 12.01
49	1156	Camphene hydrate^a^	C_10_H_18_O	154	–	–	–	–	–	–	0.23 ± 0.00	2.91 ± 0.01
50	1169	**(*Z*)-Chrysanthenol**^**a**^	C_10_H_16_O	152	–	–	–	–	–	–	0.37 ± 0.01	4.66 ± 0.02
51	1170	Pinocarvone^a^	C_10_H_14_O	150	–	–	1.26 ± 0.02	6.93 ± 0.08	–	–	3.30 ± 0.01	41.10 ± 0.02
52	1173	Borneol^e^	C_10_H_18_O	154	3.75 ± 0.04	24.41 ± 0.12	1.12 ± 0.01	6.18 ± 0.02	5.09 ± 0.03	56.82 ± 0.16	4.15 ± 0.02	51.57 ± 0.64
53	1179	Menthol^a^	C_10_H_20_O	156	–	–	–	–	–	–	0.88 ± 0.02	10.91 ± 0.04
54	1184	4-Terpineol^a^	C_10_H_18_O	154	–	–	0.40 ± 0.00	2.22 ± 0.02	0.27 ± 0.02	3.05 ± 0.04	2.36 ± 0.04	29.40 ± 0.47
55	1192	*p*-Cymen-8-ol^a^	C_10_H_14_O	150	–	–	–	–	–	–	1.12 ± 0.02	13.94 ± 0.24
56	1197	*α-*Terpineol^a^	C_10_H_18_O	154	–	–	–	–	–	–	0.54 ± 0.02	6.70 ± 0.34
57	1204	Myrtenol^a^	C_10_H_16_O	152	0.09 ± 0.00	0.60 ± 0.02	–	–	–	–	2.03 ± 0.02	25.27 ± 0.48
58	1206	Myrtenal^b^	C_10_H_14_O	150	–	–	0.27 ± 0.00	1.47 ± 0.00	–	–	–	–
59	1219	Verbenone^e^	C_10_H_14_O	150	–	–	0.27 ± 0.01	1.50 ± 0.02	0.68 ± 0.00	7.56 ± 0.02	1.06 ± 0.02	13.18 ± 0.04
60	1226	(*E*)-Carveol^c^	C_10_H_16_O	152	–	–	0.15 ± 0.00	0.84 ± 0.01	–	–	1.21 ± 0.08	15.01 ± 0.18
61	1238	(*Z*)-Carveol^a^	C_10_H_16_O	152	–	–	–	–	–	–	0.25 ± 0.04	3.14 ± 0.21
62	1250	**Cuminaldehyde**^**b**^	C_10_H_12_O	148	–	–	0.22 ± 0.00	1.20 ± 0.01	–	–	–	–
63	1253	Carvone^e^	C_10_H_14_O	150	–	–	–	–	–	–	0.27 ± 0.02	3.30 ± 0.22
64	1293	Bornyl acetate^c^	C_12_H_20_O_2_	196	3.10 ± 0.08	20.26 ± 0.24	–	–	1.92 ± 0.02	21.39 ± 0.09	–	–
65	1304	Indole^g^	C_8_H_7_N	117	–	–	–	–	–	–	0.62 ± 0.02	7.74 ± 0.42
66	1323	2-Methoxy-4-vinylphenol^a^	C_9_H_10_O_2_	150	–	–	–	–	–	–	1.61 ± 0.01	20.06 ± 0.46
67	1354	***α-*Longipinene**^**d**^	C_15_H_24_	204	0.52 ± 0.00	3.38 ± 0.06	–	–	0.72 ± 0.00	8.06 ± 0.01	–	–
68	1357	*α-*Cubebene^d^	C_15_H_24_	204	1.65 ± 0.02	10.75 ± 0.06	0.28 ± 0.00	1.62 ± 0.02	0.96 ± 0.02	10.75 ± 0.04	–	–
69	1366	Eugenol^a^	C_10_H_12_O_2_	164	–	–	–	–	–	–	0.27 ± 0.04	3.30 ± 0.06
70	1372	***α-*Ylangene**^**d**^	C_15_H_24_	204	0.44 ± 0.00	2.85 ± 0.02	–	–	0.32 ± 0.00	3.60 ± 0.01	–	–
71	1385	*α-*Copaene^d^	C_15_H_24_	204	1.50 ± 0.00	9.75 ± 0.04	1.83 ± 0.00	10.05 ± 0.01	1.68 ± 0.04	18.73 ± 0.18	2.46 ± 0.04	30.60 ± 0.44
72	1391	**(*Z*)-*β*-Elemene**^**d**^	C_15_H_24_	204	2.14 ± 0.00	13.95 ± 0.10	–	–	–	–	–	–
73	1392	Modhephene^d^	C_15_H_24_	204	–	–	0.23 ± 0.00	1.26 ± 0.00	–	–	0.59 ± 0.02	7.34 ± 0.34
74	1395	**Benzyl pentanoate**^**c**^	C_12_H_16_O_2_	192	–	–	0.26 ± 0.01	1.44 ± 0.02	–	–	–	–
75	1396	*β-*Bourbonene^d^	C_15_H_24_	204	–	–	–	–	0.42 ± 0.00	4.67 ± 0.02	–	–
76	1399	*β-*Cubebene^d^	C_15_H_24_	204	–	–	–	–	2.60 ± 0.02	28.96 ± 0.16	–	–
77	1409	(*Z*)-Jasmone^e^	C_11_H_16_O	164	–	–	–	–	–	–	0.60 ± 0.02	7.41 ± 0.30
78	1421	***α-*Gurjunene**^**d**^	C_15_H_24_	204	0.43 ± 0.00	2.82 ± 0.02	–	–	0.96 ± 0.00	10.73 ± 0.08	–	–
79	1424	*β-*Caryophyllene^d^	C_15_H_24_	204	12.08 ± 0.18	78.64 ± 0.41	5.76 ± 0.04	31.68 ± 0.12	4.61 ± 0.04	51.45 ± 0.22	7.80 ± 0.04	97.04 ± 0.33
80	1441	*β-*Copaene^d^	C_15_H_24_	204	–	–	0.22 ± 0.02	1.23 ± 0.02	1.19 ± 0.02	13.27 ± 0.06	0.59 ± 0.02	7.40 ± 0.20
81	1443	**(*Z*)-*α*-Bergamotene**^**d**^	C_15_H_24_	204	1.60 ± 0.00	10.44 ± 0.02	–	–	3.21 ± 0.04	35.82 ± 0.10	–	–
82	1444	**Aromandendrene**^**d**^	C_15_H_24_	204	0.20 ± 0.02	1.30 ± 0.08	5.02 ± 0.02	27.99 ± 0.01	–	–	–	–
83	1455	Coumarin^c^	C_9_H_6_O_2_	146	–	–	2.71 ± 0.00	14.91 ± 0.02	–	–	2.16 ± 0.02	26.88 ± 0.22
84	1456	Neryl acetone^e^	C_13_H_22_O	194	–	–	–	–	0.59 ± 0.00	6.62 ± 0.02	–	–
85	1458	**Dihydropseudoionone**^**e**^	C_13_H_22_O	194	–	–	0.14 ± 0.00	0.75 ± 0.00	–	–	–	–
86	1461	(*E*)-β-Farnesene^d^	C_15_H_24_	204	1.43 ± 0.00	9.34 ± 0.02	0.82 ± 0.01	4.53 ± 0.04	–	–	1.27 ± 0.04	15.85 ± 0.06
87	1466	***α-*Caryophyllene**^**d**^	C_15_H_24_	204	2.92 ± 0.04	19.09 ± 0.11	0.62 ± 0.00	3.42 ± 0.00	2.92 ± 0.04	32.58 ± 0.12	1.31 ± 0.02	16.31 ± 0.08
88	1468	**(*Z*)-4,5-Muuroladiene**^**d**^	C_15_H_24_	204	1.07 ± 0.00	6.98 ± 0.02	–	–	–	–	–	–
89	1471	***α-*Acoradiene**^**d**^	C_15_H_24_	204	–	–	0.17 ± 0.00	1.02 ± 0.01	–	–	–	–
90	1483	***α-*Curcumene**^**d**^	C_15_H_22_	202	3.20 ± 0.02	20.87 ± 0.06	–	–	5.75 ± 0.04	64.22 ± 0.20	–	–
91	1488	*γ-*Muurolene^d^	C_15_H_24_	204	6.49 ± 0.06	42.39 ± 0.18	–	–	1.01 ± 0.02	11.28 ± 0.08	3.79 ± 0.01	47.18 ± 0.44
92	1490	Valencene^d^	C_15_H_24_	204	–	–	–	–	5.26 ± 0.04	58.69 ± 0.18	–	–
93	1494	Germacrene D^d^	C_15_H_24_	204	23.77 ± 0.14	155.27 ± 0.44	0.43 ± 0.00	2.34 ± 0.01	–	–	2.14 ± 0.02	26.66 ± 0.24
94	1496	**(*E*)-*β*-Ionone**^**e**^	C_13_H_20_O	192	–	–	0.89 ± 0.01	4.92 ± 0.04	–	–	–	–
95	1500	*β-*Selinene^d^	C_15_H_24_	204	0.74 ± 0.02	4.83 ± 0.04	8.29 ± 0.02	46.65 ± 0.21	1.62 ± 0.02	18.10 ± 0.08	4.20 ± 0.03	52.23 ± 0.13
96	1505	*α-*Selinene^d^	C_15_H_24_	204	–	–	–	–	0.90 ± 0.00	10.05 ± 0.02	–	–
97	1506	***α-*Muurolene**^**d**^	C_15_H_24_	204	–	–	–	–	1.37 ± 0.02	15.25 ± 0.04	–	–
98	1509	*β-*Bisabolene^d^	C_15_H_24_	204	0.39 ± 0.00	2.54 ± 0.02	–	–	1.70 ± 0.02	18.96 ± 0.06	–	–
99	1514	*γ-*Cadinene^d^	C_15_H_24_	204	2.06 ± 0.00	13.46 ± 0.06	0.97 ± 0.01	5.37 ± 0.04	–	–	1.18 ± 0.01	14.71 ± 0.04
100	1517	***β-*Sesquisabinene**^**d**^	C_15_H_24_	204	–	–	–	–	1.83 ± 0.02	20.46 ± 0.06	–	–
101	1518	*δ-*Cadinene^d^	C_15_H_24_	204	4.50 ± 0.04	29.40 ± 0.20	1.61 ± 0.01	8.88 ± 0.04	2.75 ± 0.05	30.74 ± 0.10	2.47 ± 0.01	30.77 ± 0.10
102	1523	**(*E*)-Cadina-1,4-diene**^**d**^	C_15_H_24_	204	0.29 ± 0.06	1.88 ± 0.02	–	–	1.41 ± 0.02	15.68 ± 0.05	–	–
103	1529	***α-*Calacorene**^**d**^	C_15_H_20_	200	–	–	–	–	1.82 ± 0.02	20.27 ± 0.03	–	–
104	1536	(*E*)-Nerolidol^a^	C_15_H_26_O	222	0.90 ± 0.00	5.85 ± 0.04	–	–	3.04 ± 0.00	33.91 ± 0.08	–	–
105	1537	(*Z*)-Nerolidol^a^	C_15_H_26_O	222	–	–	–	–	–	–	1.67 ± 0.02	20.80 ± 0.04
106	1553	Caryophyllene oxide^f^	C_15_H_24_O	220	3.31 ± 0.04	21.61 ± 0.10	31.9 ± 0.11	186.48 ± 6.21	9.42 ± 0.02	105.12 ± 0.22	18.83 ± 0.14	234.16 ± 8.23
107	1564	Germacrene B^d^	C_15_H_24_	204	0.16 ± 0.00	1.07 ± 0.02	–	–	–	–	–	–
108	1570	**Junenol**^**a**^	C_15_H_26_O	222	–	–	–	–	2.88 ± 0.04	32.19 ± 0.08	–	–
109	1571	***α-*Guaiol**^**a**^	C_15_H_26_O	222	–	–	–	–	4.05 ± 0.06	45.25 ± 0.06	–	–
110	1576	**Palustrol**^**a**^	C_15_H_26_O	222	0.27 ± 0.00	1.73 ± 0.01	–	–	–	–	–	–
111	1578	Spathulenol^a^	C_15_H_24_O	220	6.13 ± 0.10	40.04 ± 0.22	–	–	12.45 ± 0.08	138.92 ± 0.42	–	–
112	1610	Salvial-4(14)-en-1-one^e^	C_15_H_24_O	220	1.81 ± 0.08	11.84 ± 0.20	2.58 ± 0.02	14.22 ± 0.06	–	–	–	–
113	1616	*α-*Humuleneepoxide II^f^	C_15_H_24_O	220	0.31 ± 0.00	2.05 ± 0.02	–	–	–	–	–	–
114	1626	**Alloaromadendrene epoxide**^**f**^	C_15_H_24_O	220	–	–	5.33 ± 0.08	29.31 ± 0.04	–	–	–	–
115	1649	**4-Cadinen-7-ol**^**a**^	C_15_H_26_O	222	–	–	3.10 ± 0.02	17.01 ± 0.06	–	–	–	–
116	1847	**Phytone**^**e**^	C_18_H_36_O	268	–	–	5.02 ± 0.02	27.99 ± 0.11	–	–	–	–
		Total			100.00	651.53	100.00	566.10	100.00	1122.73	100.00	1237.79

Bold mark compounds name = Firstly reported volatile organic compounds in this study,– = Not detected

^a^ Alcohol

^b^ Aldehyde

^c^Ester

^d^Hydrocarbon

^e^ Ketone

^f^ Ether

^g^Miscellaneous

Data were reported by mean ± standard deviation (n = 3); 0.00 = The value is less than 0.01, ^*^RI Retention index, ^**^MF Molecular formula, ^***^MW Molecular weight, *I.S*. Internal standard;

In the current analysis, leaves of both plants were extracted for volatile compounds by HS-SPME and SDE. The extracts were analyzed by GC/MS, which identified more compounds compared to already reported studies (Haghighian et al., 2008). A total of 116 volatile organic compounds were identified from the subject leaves and this study thus reported 33 volatile organic compounds for the first time in comparison to published literature (Table 1, marked bold). The total amount of volatile organic compounds for *A. artemisiifolia* L. leaves and *A. annua* L. leaves ranged from 651.53 mg/kg to 1,122.73 mg/kg and 566.1 mg/kg to 1,237.79 mg/kg through HS-SPME and SDE techniques, respectively.

### Volatile compounds of *A. artemisiifolia* L. leaves by HS-SPME technique

A total of 46 volatile organic compounds were identified in *A. artemisiifolia* L. leaves amounting 651.53 mg/kg (Table 1). These included 28 hydrocarbons (76.49%, 499.20 mg/kg), 7 alcohols (11.62%, 75.81 mg/kg), 4 ketones (3.92%, 25.59 mg/kg), 3 ethers (4.09%, 26.70 mg/kg), 3 aldehydes (0.61%, 3.97 mg/kg), and 1 ester (3.1%, 20.26 mg/kg) (Table 2). The terpene group compounds (99.12%, 646.91 mg/kg) were the maximum in *A. artemisiifolia* L. leaves; which included 21 sesquiterpene hydrocarbons (67.58%, 441.00 mg/kg), 6 oxygenated sesquiterpenes (12.73%, 83.12 mg/kg), 8 oxygenated monoterpenes (9.9%, 64.59 mg/kg), and 7 monoterpene hydrocarbons (8.91%, 58.20 mg/kg) (Table 3). The highest concentration of volatile organic compounds were of *β-*caryophyllene (12.08%, 78.64 mg/kg), followed by γ-muurolene (6.49%, 42.39 mg/kg), spathulenol (6.13%, 40.04 mg/kg), and *δ*-cadinene (4.5%, 29.40 mg/kg). *A. artemisiifolia* L. leaves gave 6 compounds including phenyl acetaldehyde, (*E*)-*β*-ocimene, artemisia alcohol, germacerene B, palustrol, and *α*-humuleneepoxide II by HS-SPME which were not detected by SDE extraction (Table 1).

**Table 2:** Relative contents of the functional groups in the volatile organic compound detected between *Ambrosia artemisiifolia* L. leaves and *Artemisia annua* L. leaves by HS-SPME and SDE techniques

Functional groups	HS-SPME						SDE					
	*Ambrosia artemisiifolia* L.			Artemisia annua L.			Ambrosia artemisiifolia L.			Artemisia annua L.		
	No	Area (%)	Amount (mg/kg)	No	Area (%)	Amount (mg/kg)	No	Area (%)	Amount (mg/kg)	No	Area (%)	Amount (mg/kg)
Alcohol	7	11.62	75.81	7	8.48	37.92	12	32.29	360.52	26	26.47	323.25
Aldehyde	3	0.61	3.97	5	1.19	6.57	5	3.42	38.16	11	1.87	23.41
Ester	1	3.1	20.26	2	2.97	16.35	1	1.92	21.39	1	2.16	26.88
Hydrocarbon	28	76.49	499.2	17	27.67	153.84	26	51.76	577.58	22	31.35	390.32
Ketone	4	3.92	25.59	7	21.96	122.07	4	1.79	19.96	7	16.01	199.05
Ether	3	4.09	26.7	3	39.69	229.35	1	9.42	105.12	3	21.49	267.14
Miscellaneous	–	–	–	–	–	–	–	–	–	1	0.62	7.74
Total	46	100	651.53	41	100	566.1	49	100	1,122.73	71	100	1,237.79

**Table 3:** Relative contents of the terpene compound in the volatile organic compound detected between *Ambrosia artemisiifolia* L. leaves and *Artemisia annua* L. leaves by HS-SPME and SDE techniques

Terpene groups	HS-SPME						SDE					
	*Ambrosia artemisiifolia* L.			*Artemisia annua* L.			Ambrosia artemisiifolia L.			Artemisia annua L.		
	No	Area (%)	Amount (mg/kg)	No	Area (%)	Amount (mg/kg)	No	Area (%)	Amount (mg/kg)	No	Area (%)	Amount (mg/kg)
Monoterpene hydrocarbons	7	8.91	58.2	4	1.42	7.80	4	6.75	75.26	11	3.55	44.23
Oxygenated monoterpene	8	9.9	64.59	13	21.66	111.33	6	11.07	123.61	23	40.17	493.56
Sesquiterpene hydrocarbons	21	67.58	441	13	26.25	146.04	22	45.01	502.32	11	27.80	346.09
Oxygenated sesquiterpene	6	12.73	83.12	5	47.93	275.01	5	31.84	355.39	2	20.50	254.96
Total	42	99.12	646.91	35	97.26	540.18	37	94.67	1,056.58	47	92.02	1,138.84

### Volatile compounds of *A. annua* L. leaves by HS-SPME technique

From Table 1, the amount of volatile organic compounds from *A. annua* L. leaves was found as 566.10 mg/kg by HS-SPME method. These included a total of 41 volatile organic compounds, with 17 hydrocarbons (27.67%, 153.84 mg/kg), 7 ketones (21.96%, 122.07 mg/kg), 7 alcohols (8.48%, 37.92 mg/kg), 5 aldehydes (1.19%, 6.57 mg/kg), 3 ethers (39.96%, 229.35 mg/kg), and 2 esters (2.97%, 16.35 mg/kg). As per Table 3, terpene group compounds were having the highest concentration (97.26%, 540.18 mg/kg), which consisted of 5 oxygenated sesquiterpenes (47.93%, 275.01 mg/kg), 13 sesquiterpene hydrocarbons (26.25%, 146.04 mg/kg), 13 oxygenated monoterpenes (21.66%, 111.33 mg/kg), and 4 monoterpene hydrocarbons (1.42%, 7.80 mg/kg). The major volatile organic compounds were caryophyllene oxide (31.90%, 186.48 mg/kg) followed by camphor (11.80%, 65.76 mg/kg), *β-*selinene (8.29%, 46.65 mg/kg), *β-*caryophyllene (5.76%, 31.68 mg/kg), alloaromadendrene epoxide (5.33%, 29.31 mg/kg), phytone (5.02%, 27.99 mg/kg), and aromandendrene (5.02%, 27.99 mg/kg). The HS-SPME method found 6 compounds such as cuminaldehyde, benzyl pentanoate, *α*-acoradiene, alloaromadendrene epoxide, 4-cadinen-7-ol, and phytone, which were not detected by SDE analysis (Table 2). In literature other studies have also reported artemisia ketone, yomogi alcohol, camphor, and *β*-selinene in *A. annua* L. leaves (Nekoei et al., 2012; Reale et al., 2011). Thus current research findings are line with the published research studies.

### Volatile compounds of *A. artemisiifolia* L. leaves by SDE technique

The amount of volatile organic compounds in *A. artemisiifolia* L. leaves by SDE extraction was 1,122.73 mg/kg. A total of 49 volatile organic compounds were reported (Table 1), belonging to different chemical groups such as 26 hydrocarbons (51.76%, 577.58 mg/kg), 12 alcohols (32.29%, 360.52 mg/kg), 5 aldehydes (3.42%, 38.16 mg/kg), 4 ketones (1.79%, 19.96 mg/kg), 1 ether (9.42%, 105.12 mg/kg), and 1 ester (1.92%, 21.39 mg/kg). The terpene group (94.67%, 1,056.58 mg/kg) was the major class of volatile organic compounds in *A. artemisiifolia* L. leaves, with 22 sesquiterpene hydrocarbons (45.01%, 502.32 mg/kg), 5 oxygenated sesquiterpenes (31.84%, 355.39 mg/kg), 6 oxygenated monoterpenes (11.07%, 123.61 mg/kg), and 37 monoterpene hydrocarbons (6.75%, 75.26 mg/kg) (Table 3). The major volatile organic compounds detected were spathulenol (12.45%, 138.92 mg/kg), caryophyllene oxide (9.42%, 105.12 mg/kg), *α*-curcumene (5.75%, 64.22 mg/kg), valencene (5.26%, 58.96 mg/kg), borneol (5.09%, 56.82 mg/kg), and *β-*caryophyllene (4.61%, 51.45 mg/kg). *A. artemisiifolia* L. leaves were found to have 12 volatile organic compounds such as sulcatone, benzene acetaldehyde, linalool, *β*-bourbonene, *β*-cubebene, neryl acetone, *β*-sesqui sabinene, junenol, and *α*-guaiol which were reported by SDE and not by HS-SPME technique (Table 1). Wang et al., (2006) have reported by steam distillation method that the germacerene D, limonene, and *α*-pinene are present in *A. artemisiifolia* L. leaves.

### Volatile compounds of *A. annua* L. leaves by SDE extraction

As shown in Table 1, the amount of volatile organic compounds in *A. annua* L. leaves was 1,237.79 mg/kg by SDE method. These were 71 compounds belonging to specific chemical functional groups including 26 alcohols (26.47%, 323.25 mg/kg), 22 hydrocarbons (31.35%, 390.32 mg/kg), 11 aldehydes (1.87%, 23.41 mg/kg), 7 ketones (16.01%, 199.05 mg/kg), 3 ethers (21.49%, 267.14 mg/kg), 1 ester (2.16%, 26.88 mg/kg). The highest proportion was of terpene group compounds (92.02%, 1,138.84 mg/kg) which included 23 oxygenated monoterpenes, 11 sesquiterpene hydrocarbons, 2 oxygenated sesquiterpenes, and 11 monoterpene hydrocarbons having 40.17%, 493.56 mg/kg; 27.80%, 346.09 mg/kg; 20.50%, 254.96 mg/kg and 3.55%, 44.23 mg/kg, respectively (Table 3). The major volatile organic compounds were caryophyllene oxide (18.83%, 234.16 mg/kg) followed by camphor (10.66%, 132.61 mg/kg), *β-*caryophyllene (7.80%, 97.04 mg/kg), (*E*)-pinocarveol (5.27%, 65.55 mg/kg), and *β-*selinene (4.20%, 52.23 mg/kg). In the current study, 14 volatile organic compounds were found only by SDE compared to HS-SPME method. Both extractions of *A. artemisiifolia* L. leaves identified compounds such as *γ*-terpinene, phenethyl alcohol, *α*-campholenal, camphene hydrate, (*Z*)-chrysanthenol, *p*-cymen-8-ol, *α*-terpineol, (*Z*)-carveol, indole, 2-methoxy-4-vinylphenol, eugenol, (*Z*)-jasmone, and (*Z*)-nerolidol (Table 1). In literature, similar compounds such as erythritol, champhore, germacrene D, artemisia ketone, *α*-caryophillene, *α*-cuvebene have already been detected by steam distillation and hydrodistillation in *A. artemisiifolia* L. leaves (Bilia et al., 2008; Haghighian et al., 2008; Juteau et al., 2002; Tzenkova et al., 2010; Vidic et al., 2018).

### Comparison of extraction techniques and identification marker compounds

Headspace-solid phase micro extraction (HS-SPME) is used to extract volatile organic compounds with short time and to extract more volatile monoterpenes. Simultaneous distilled extraction (SDE) is capable of extracting higher amount of volatile organic compounds and requires longer extraction time at high temperature. Both techniques have been used for volatile compounds in the past around the world (Majcher and Jeleń, 2009). Some researchers have compared HS-SPME with other conventional methods and have reported that the HS-SPME method as more significant than other traditional methods for the determination of volatile organic compounds in herbs (Majcher and Jeleń 2009; Yang et al., 2011). The SDE technique may subsequently increase the low volatile organic compounds with a high molecular weight, such as straight-chain acids, and sesquiterpenes.

In the current study, 116 volatile organic compounds were reported, with 11 compounds (hexanal, *α*-pinene, benzaldehyde, limonene, borneol, *α*-copaene, *β*-cayophyllene, *α*-caryophyllene, *β*-selinene, *δ*-cadinene, and caryophyllene oxide) identified in the leaves of both species applying the 2 extraction methods. From Table 3, the sesquiterpene hydrocarbons (67.58%, 441 mg/kg) were relatively in higher content in *A. artemisiifolia* L. leaves whereas oxygenated sesquiterpenes (47.93%, 275.01 mg/kg) were detected in higher concentrations in *A. annua* L. leaves by HS-SPME technique. Both HS-SPME and SDE methods extracted more terpene hydrocarbons from *A. artemisiifolia* L. leaves and oxygenated terpene compounds from *A. anuua* L. leaves.

Volatile organic compounds are liable for flavour, aroma and bioactivity. In addition, these have importance for chemical fingerprinting. The chemical elements have various forms of plant essential oils, which are regularly used to identify the variety or to improve the chemotypes. In the current study, both leaves were found to consist of mainly hydrocarbons, ethers, and alcoholic type of volatile organic compounds.

Each country and possibly each region may have a distinct type of *A. annua* L. leaves and *A. artemisiifolia* L. leaves essential oil, called chemotypes. Different chemotypes such as erythritol, champhore, germacrene D, artemisia ketone, *α*-caryophillene, and *α*-cuvebene have been reported for most of the *A. annua* L. leaves essential oil in literature (Bilia et al., 2008; Haghighian et al., 2008; Juteau et al., 2002; Tzenkova et al., 2010; Vidic et al., 2018; Wang et al., 2006). In this study, spathulenol, caryophyllene oxide, and *β*-caryophyllene chemotypes were observed in *A. artemisiifolia* L. leaves whereas caryophyllene oxide, champhor, and *β*-selinene chemotypes were detected in *A. annua* L. leaves. This variation may be due to the geographical and ecological conditions, time of harvesting, and age of the plant (Bagamboula et al., 2004).

In conclusion, this study determined the volatile organic compounds from *A. artemisiifolia* L. leaves and *A. annua* L. leaves using HS-SPME and SDE extraction methods coupled with GC/MS analysis. The results demonstrated remarkable differences in identified volatile organic compounds concentration in both leaves by 2 extraction methods. The highest concentration of volatile organic compounds was detected in *A. annua* L. leaves (1,237.79 mg/kg) by SDE technique whereas the lowest value was observed in the same leaves (566.1 mg/kg) by HS-SPME extraction. A total of 116 volatile components were identified, extracted by SDE compared to 71 volatile organic compounds by HS-SPME technique, while 33 volatile organic compounds were detected for the first time compared to published literature on *A. artemisiifolia* L. leaves and *A. annua* L. leaves. The common major volatile organic compounds (caryophyllene oxide and champhor) were detected in leaves of both samples. The identified marker compounds in *A. artemisiifolia* L were *γ*-muurolene and spathulenol while *β*-selinene and caryophyllene were reported in *A. annua* L. leaves. Thus, the present findings extend the characterization of volatile organic compounds of *A. annua* L. leaves and *A. artemisiifolia* L. leaves and reported some distinguishing compounds which may be used for their characterization.
